# SARS-CoV-2 Ig G among Healthcare Workers and the General Population

**DOI:** 10.3390/pathogens10040465

**Published:** 2021-04-12

**Authors:** Gregorio P. Milani, Mario G. Bianchetti, Giuseppe Togni, Andreas W. Schoenenberger, Franco Muggli

**Affiliations:** 1 Istituto Pediatrico della Svizzera Italiana, 6500 Bellinzona, Switzerland; 2 Fondazione IRCCS Ca’ Granda Ospedale Maggiore Policlinico, 20122 Milan, Italy; 3 Department of Clinical Sciences and Community Health, University of Milan, 20122 Milan, Italy; 4 Faculty of Biomedical Science, Università della Svizzera Italiana, 6900 Lugano, Switzerland; mario.bianchetti@usi.ch (M.G.B.); fmuggli@bluewin.ch (F.M.); 5 Microbiology Laboratory, Unilabs, 1296 Coppet, Switzerland; giuseppe.togni@unilabs.com; 6 Department of Geriatrics, Inselspital, Bern University Hospital, University of Bern, 3010 Bern, Switzerland; andreas.schoenenberger@stgag.ch

**Keywords:** COVID19, SARS-CoV-2, infection risk, healthcare providers, physicians

## Abstract

It is assumed that healthcare workers are at the highest risk to be infected by severe acute respiratory syndrome coronavirus 2 (SARS-CoV-2). However, few data from healthcare workers who do not primarily take care of patients with SARS-CoV-2 infection support this assumption. We investigated the prevalence of immunoglobulin G (Ig G) against SARS-CoV-2 among healthcare workers who do not primarily take care of patients with SARS-CoV-2 infection and the general population in a well-defined geographical area. The first part of the study was conducted in May 2020 in Val Mesolcina (Southern Switzerland), a valley with ~8000 inhabitants. All healthcare workers were invited. All participants (*n* = 488) of the Swiss Longitudinal Cohort Study (SWICOS), a cohort representative of the general population, were also invited. Circulating Ig G against spike protein subunit 1 of SARS-CoV-2 were tested in each subject. Subjects with positive Ig G were tested again after 6 months. The condition of being a healthcare worker, rather than a part of the general population, was tested as a predictor of seroprevalence positivity by both simple and multiple (adjusted for age and sex) logistic regression. Eleven (2.6%) of the 423 SWICOS participants and 46 (16%) out of 289 healthcare workers were positive for antibodies against SARS-CoV-2. The seroprevalence OR was 7.01 (95% CI: 3.53–15.47) for healthcare workers as compared to SWICOS participants. After adjusting for age and gender, the seroprevalence OR was 5.13 (95% CI: 2.54–10.40). About three quarters of the subjects in the SWICOS (73%) and in healthcare (79%) group with a previous positive serology still presented positive Ig G against the SARS-CoV-2 after 6 months. The present seroprevalence data point out that the SARS-CoV-2 infection is seven times higher among healthcare workers than in the general population of Val Mesolcina. Efforts to effectively protect all the healthcare personnel are needed.

## 1. Introduction

Severe acute respiratory syndrome coronavirus 2 (SARS-CoV-2) is mainly transmitted by air droplets [1]. It is therefore assumed the healthcare workers are at the highest risk to be infected [2]. Although this assumption was not always confirmed and a few reports observed a rather low prevalence of seroconversion among health personnel [3,4], most investigations reported a relevant incidence of infection among healthcare workers who care patients affected with (COVID19) [5,6]. Limited data are available on health care providers who do not primary take care of COVID19 patients [2]. The aim of this study was to compare the coronavirus disease 19 prevalence of Ig G antibodies against SARS-CoV-2 of healthcare workers, who do not primary take care of COVID19 patients, with those of the general population in a well-defined geographical area during the first wave of the pandemic in spring 2020. The secondary aim was to investigate the persistence of antibodies in subjects with a positive serology.

## 2. Results

### 2.1. Study Participants

A total of 423 (87%) out of the 488 Swiss Longitudinal Cohort Study (SWICOS) participants and 289 (84%) out of the 344 healthcare workers of Val Mesolcina volunteered to participate. Demographics, comorbidities and relevant drug treatments of the study participants are given in Table 1. The SWICOS participants were older and more frequently male than healthcare workers.

Arterial hypertension, cardiovascular diseases, and medication with immunosuppressants, ACE-inhibitors or sartans were more common among SWICOS participants.

### 2.2. Prevalence of IgG against SARS-CoV-2 and Clinical History

Only 11 (2.6%) out of the 423 SWICOS participants were positive for Ig G against SARS-CoV-2. This figure was relevantly higher (*p* < 0.0001) for healthcare workers: a total of 46 (16%) out of 289 were positive for antibodies against SARS-CoV-2. Among the 46 healthcare workers with anti-SARS-CoV-2 Ig G, 31 were active in long-term care facilities for elderly, 12 in outpatient clinics and 3 in home care services (Table 2). They were 23 paramedics, 14 nurses, 6 physicians and 3 student paramedics.

Upper respiratory symptoms, anosmia, fever, asthenia, and muscle ache were more frequently reported by the 56 subjects with antibodies against SARS-CoV-2 as compared to the 656 subjects without. The tendency was on the average similar in healthcare workers and in the general population.

### 2.3. Clinical History and Lifestyle Characteristics of Subjects with IgG against SARS-CoV-2

The prevalence of at least one comorbidity, and medications with immunosuppressants, ACE-inhibitors or sartans was similar among SWICOS participants and healthcare workers with Ig G anti-SARS-CoV-2 (Table 2). Similarly, smoking habits were not different (*p* = 0.2) in SWICOS participants (previous smokers, *n* = 4; non-smokers, *n* = 7) and healthcare workers (current smokers, *n* = 10; previous smokers, *n* = 9; non-smokers, *n* = 27) with Ig G anti-SARS-CoV-2. On the contrary, SWICOS participants were more frequently users of public transports as compared with healthcare workers (4 out 11 vs 3 out 46, respectively, *p* < 0.05). 

The seroprevalence OR for healthcare workers as compared to SWICOS participants was 7.01. After adjusting for age and gender, the seroprevalence OR was slightly lower (5.13), as shown in Table 3.

### 2.4. Follow-Up Serology of Subjects with Ig G Anti-SARS-CoV-2

All SWICOS participants (*n* = 11) and 39 out of 46 healthcare workers with positive Ig G against SARS-CoV-2 at baseline participated in the follow-up examination: a total of 8 (72%) out 11 and 31 (79%) out of 39 subjects still presented Ig G against SARS-CoV-2. The frequency was similar in the two groups (*p* = 0.49).

## 3. Discussion

The present seroprevalence study points out that the SARS-CoV-2 infection is seven times higher among healthcare workers than in the general population of Val Mesolcina. In both groups, symptoms consistent with SARS-CoV-2 infection were more common among subjects with Ig G antibodies as compared to subjects without. Moreover, antibodies persist at 6 months in approximately three quarters of the cases (with a similar figure in both study groups).

Healthcare workers of Val Mesolcina do not primarily take care of acute SARS-CoV-2 patients, because these patients are managed in other hospitals of Southern Switzerland. Hence, we speculate that the contact with undiagnosed, mildly symptomatic subjects in outpatient clinics or outbreaks in long-term care facilities might have played a significant role in SARS-CoV-2 infection in this group of workers. The finding that the healthcare workers with Ig G anti SARS-CoV-2 used less frequently public transports, a possible source of SARS-CoV-2 infection [7], further supports the mentioned hypothesis. 

Smokers have been considered at lower risk of SARS-CoV-2 infection, but at higher risk of a severe disease [8]. In our rather small sample, no difference in smoking habits was found between healthcare workers and the general population with Ig G against SARS-CoV-2, thus precluding a major role of smoking in SARS-CoV-2 susceptibility. Furthermore, no difference was observed between the two populations, regarding the presence of at least one comorbidity and medications with immunosuppressants, ACE-inhibitors or sartans, three further factors possibly associated with the risk of infection [9].

Studies conducted in hospital-based settings found an increased risk of SARS-CoV-2 infection among healthcare workers. In a survey conducted in UK and USA, healthcare workers managing COVID19 inpatients were more frequently infected by SARS-CoV-2 (hazard ratio of 3.40, 95% CI 3.37–3.43) as compared with the general community [10]. A study including a cohort of healthcare workers active in two USA university hospitals and a cohort of university members not active in healthcare reported a 7.0% (95% CI 4.7–9.3) greater absolute risk of SARS-CoV-2 infection, as documented by oropharyngeal swabs, in the first cohort [11]. Taken together, the results of these investigations and those of our study point out that the risk of SARS-CoV-2 infection is markedly increased among all healthcare workers.

Using the laboratory assay applied for our study, the seroprevalence in healthcare workers of a tertiary hospital in Germany was 1.6 % [12]. The difference with our data may result from the very high burden of SARS-CoV-2 observed in Southern Switzerland during the first wave pandemic [4]. Moreover, healthcare providers working in SARS-CoV-2-free departments in Switzerland do not dress N95 masks but only surgical masks [13], which provide a limited protection against this virus [14,15]. Therefore, insufficient personal protective measures might have also played a role in facilitating the virus spread among healthcare workers. Since healthcare workers face every day the consequences of this unexpected infectious threat, often with poor resources and in stressful conditions, new efforts should be made to support this category [16].

The rather high frequency of subjects with persistent Ig G at 6 months is likely related to the fact that the vast majority of them had a previous history with symptoms consistent with SARS-CoV-2 infection. However, the relevance of persisting Ig G levels is still matter of debate [17,18,19].

This study has both limitations and strengths. It was performed with an Ig G assay against the SARS-CoV-2, which is validated and reliable for population studies [20]. Regrettably, we did not measure Ig M or Ig A. However, the role of Ig M and Ig A in this condition is still to be elucidated. Moreover, we did not track possible infections among family members and other contacts. The main strength is that this study was conducted in a well-defined geographical area.

In conclusion, this study suggests that healthcare workers who do not take care of COVID19 inpatients are five times more at risk of SARS-CoV-2 infection than the general population. Efforts to more effectively protect and support all the healthcare personnel are needed for the current coronavirus pandemic and for any possible new emergency.

## 4. Materials and Methods

The first part of this study was conducted between May 15 and 31, 2020 in Val Mesolcina, an alpine valley in Southern Switzerland with ~8′000 inhabitants. All subjects of the Swiss Longitudinal Cohort Study, a prospective investigation involving 488 subjects representative of the general population of the valley [21], were invited to participate the study by mail. Moreover, healthcare workers active in long-term care facilities, outpatient clinics, homecare or ambulance services of the valley were asked to participate through the heads of the healthcare structures or services. Healthcare workers directly involved in the management of COVID19 inpatients were not included, because no institution responsible for the care of this condition is present in Val Mesolcina. After written consent, the participants filled-in a structured questionnaire. The questionnaire addressed the following issues: (1) demographics (age and gender), (2) clinical history from January 2020, comprising symptoms consistent with SARS-CoV-2 infection, comorbidities (diabetes mellitus, chronic obstructive pulmonary disease or asthma, arterial hypertension, cardiovascular diseases, autoimmune diseases, cancer), medication with immunosuppressants, ACE-inhibitors or sartans and, finally (3) current lifestyle, including smoking habit (current smoker, previous smoker, non-smoker) and the use of public transports (≥3 times per week). Finally, blood was drawn to detect specific Ig G against spike protein subunit 1 of SARS-CoV-2 using an enzyme-linked immunosorbent assay (Euroimmun Medizinische Labordiagnostika, Lübeck, Germany) [20]. The cut-off value for positivity was >1.1 as recommended by the manufacturer [22]. The questionnaire and the blood tests were performed in the same period for both the groups. 

In the second part of the study, performed 6 months later, blood was collected for a new serology in subjects (both SWICOS participants and healthcare workers) found to be positive for Ig G in the first part of the study. 

Continuous data are presented as mean (±standard deviation) and were analyzed using the two-tailed *t*-test. Categoric data are presented as absolute number (and percentage) and were analyzed using the Fisher test. The condition to be a healthcare worker, rather than to be part of the general population, was tested as a predictor of seroprevalence positivity by a simple logistic regression. Anticipating a possible difference in demographics between the healthcare worker and the general population group, the results of the logistic regression were also adjusted for age and gender. The Ethics Committee of Nordwest-und Zentralschweiz approved this study (EKNZ 2014-209).

## Figures and Tables

**Table 1:** Characteristics of the healthcare workers and Swiss Longitudinal Cohort Study SWICOS participants included in the study.

Charachteristics	All	Healthcare Workers	SWICOS Participants
*n*	712	289	423
Age (years), mean ± SD	50 ± 16	44 ± 15 **	53 ± 16
Female gender, *n* (%)	437 (61)	202 (70) **	235 (56)
Comorbidities, *n* (%)			
Diabetes mellitus	17 (2.4)	4 (1.4)	13 (3.1)
*COPD* ^&^ *or asthma*	32 (4.5)	7 (2.4)	25 (5.9)
*Arterial hypertension*	73 (10)	15 (5.2) **	58 (14)
*Cardiovascular diseases*	28 (3.9)	3 (1.0) **	25 (5.9)
*Autoimmune diseases*	12 (1.7)	4 (1.4)	8 (1.9)
*Cancer*	15 (2.1)	3 (1.0)	12 (2.8)
No comorbidity, *n* (%)	571 (80)	259 (90) ***	312 (74)
Relevant drug treatment, *n* (%)			
*Immunosuppressants* ^^^	41 (5.8)	7 (2.4) **	34 (8.0)
*ACE-inhibitors or sartans*	48 (6.7)	11 (3.8) *	37 (8.7)

^&^ Chronic obstructive pulmonary disease; ^^^ including systemic corticosteroids. * = *p* < 0.05, ** = *p* < 0.002, *** = *p* < 0.0001.

**Table 2:** Demographic data, detection of SARS-CoV-2 Ig G at baseline and clinical history from January 2020 of healthcare workers and SWICOS participants.

Variables	All		Healthcare Workers		SWICOS Participants	
	Ig G Positive	Ig G Negative	Ig G Positive	Ig G Negative	Ig G Positive	Ig G Negative
*n*	57	655	46	243	11	412
Age (years), mean ± SD	41 *** ± 15	50 ± 17	40 ± 16	44 ± 15	47 ± 6.9	53 ± 17
Gender, female, *n* (%)	41 (73)	398 (61)	31 (67)	171 (70)	8 (73)	227 (55)
At least one comorbidity, *n* (%)	9 (16)	132 (20)	7 (15)	23 (9.5)	2 (18)	109 (26)
Immunosuppressants, *n* (%)	4 (7.0)	37 (5.6)	3 (6.5)	4 (8.2)	1 (9.1)	33 (8.0)
ACE-inhibitors or sartans, *n* (%)	3 (5.3)	45 (6.9)	2 (4.3)	9 (3.7)	1 (9.1)	36 (8.7)
Clinical History, *n* (%)						
*Upper respiratory symptoms*	49 *** (86)	346 (53)	43 *** (93)	126 (52)	6 (55)	220 (55)
*Lower respiratory symptoms*	6 (11)	31 (4.7)	6 * (13)	12 (4.9)	0 (0.0)	19 (4.6)
*Anosmia*	32 *** (57)	30 (4.6)	28 *** (61)	7 (2.9)	4 * (36)	23 (5.6)
*Diarrhea*	17 (30)	124 (19)	13 (28)	41 (17)	4 (36)	83 (20)
*Fever*	26 *** (46)	112 (17)	20 ** (43)	49 (20)	6 ** (55)	63 (15)
*Asthenia*	42 *** (74)	280 (43)	34 *** (74)	92 (38)	8 (73)	188 (46)
*Muscle ache*	25 *** (44)	179 (27)	18 (39)	64 (26)	7 * (63)	115 (28)

* = *p* < 0.05, ** = *p* < 0.002, *** = *p* < 0.0001, versus IgG negative.

**Table 3:** Odds ratio (ORs) of presenting Ig G against SARS-CoV-2 and corresponding 95% confidence interval (95% CI) among healthcare workers compared with the general population (univariate and multiple logistic regression).

Type of Model	ORs	95% CI	*p* Value
Unadjusted model	7.01	3.53–15.47	<0.001
Adjusted model *	5.13	2.54–10.40	<0.001

* Age and gender were used for the adjustment.

## Data Availability

Data are available upon reasonable request to the corresponding author.
